# Trends and variation in the management of oesophagogastric cancer patients: a population-based survey

**DOI:** 10.1186/1472-6963-9-231

**Published:** 2009-12-15

**Authors:** Georgios Lyratzopoulos, Josephine M Barbiere, Chetna Gajperia, Michael Rhodes, David C Greenberg, Karen A Wright

**Affiliations:** 1 Department of Public Health and Primary Care, University of Cambridge School of Clinical Medicine, Institute of Public Health, Forvie Site, Robinson Way, Cambridge, CB2 0SR, UK; 2 Norfolk and Norwich University Hospital, Colney Lane, Norwich, NR4 7UY, UK; 3 Eastern Cancer Registration and Information Centre, Unit C - Magog Court, Shelford Bottom, Cambridge, CB22 3AD, UK

## Abstract

**Background:**

Previous evidence indicates potential variation in the quality of care of cancer patients. We aimed to examine whether recent changes in the treatment of oesophagogastric cancers have been distributed equally among different patient subgroups.

**Methods:**

We analysed population-based cancer registry data about the treatment patterning of oesophagogastric cancer (other than oesophageal squamous cell carcinoma) during 1995-2006.

**Results:**

There were 14,077 patients aged ≥40 years (69% men). There was only limited information on stage, and no information on co-morbidity status. During successive triennia, curative surgery use decreased from 28% to 20% (p < 0.001) whilst chemotherapy use increased from 9% to 30% (p < 0.001). Use of palliative surgery and of radiotherapy increased significantly but modestly (7% to 10%, and 9% to 11%, respectively). In multivariable logistic regression adjusting for age group, gender, diagnosis period and tumour type, curative surgery and chemotherapy were used less frequently in more deprived patients [per increasing deprivation group Odds Ratio (OR) = 0.96, 95% Confidence Interval (CI) 0.93-0.99, and OR = 0.90, 95%CI 0.87-0.93, respectively, p < 0.001 for both)]. Chemotherapy was also used less frequently in women (OR = 0.76, p < 0.001).

**Conclusions:**

During the study period, curative surgery decreased by a third and chemotherapy use increased by more than three-fold, reflecting improvements in the appropriateness and quality of management, but chemotherapy use, in particular, was unequal, both by socioeconomic status and gender.

## Background

Oesophageal and stomach cancers [including Oesophageal Squamous Cell Carcinoma (OSCC), Oesophageal Adeno-Carcinoma (OAC), Junctional/Cardia Adenocarcinoma (JCA) and Non-Cardia Gastric Adenocarcinoma (NCGA)] are common. In the UK, they account for over 15,000 new cases per year, and over 13,000 deaths, with stomach and oesophageal cancers being the 6th and 8th most common malignancies in men[1]. Although radical surgery could offer cure, fewer than a quarter of all patients present at a stage that can be treated surgically[2]. For suitable patients, peri-operative chemotherapy has been shown to improve survival[3,4]. This is thought to have led to a substantial increase in chemotherapy use in recent years[5].

In recent periods, five-year survival has been <17% for stomach cancer, and <13% for oesophageal cancer[6-8]. In men with oesophagogastric cancer, survival has been substantially poorer in more socio-economically deprived patients[5,7-10]. In contrast, in England and Wales, five-year relative survival differences between most and least deprived women were relatively small (-0.2%) for oesophageal cancer,[8] whereas five-year survival was better (+1.7%) in most compared with least deprived women for stomach cancer[7]. The exact causes of socioeconomic and gender differences in survival are not known, but in theory those may reflect differences in clinical management, tumour type or stage at presentation, and co-morbidity. Differences in patient (e.g. co-morbidity) or tumour factors and stage do not seem to fully explain socioeconomic inequalities[11-15]. Conversely, differences in healthcare may be responsible,[16] and, in some studies, no survival inequalities were observed when patients were treated in the same service setting[12,17,18]. However, overall, the evidence supporting any of the above explanations is limited[13]. A number of high profile policy initiatives over recent years in the UK have aimed to focus efforts on improving outcomes for cancer patients, and on reducing socioeconomic inequalities in outcomes[19,20].

Motivated by the above considerations, we conducted a study to examine whether recent changes in the treatment of oesophagogastric cancers have been distributed equally among different patient subgroups.

## Methods

Non-identifiable information was obtained from the Eastern Cancer Registration and Information Centre (ECRIC), one of the regional English population-based cancer registries, responsible for a general population of about 5.5 million (which is about 10% of the English population) residing in the East of England Government Office Region, including the counties of Essex, Bedfordshire, Hertfordshire, Cambridgeshire, Norfolk and Suffolk and the Unitary Authorities of Luton and of Peterborough. The Registry's proportion of 'Death Certificate Only' registrations for all malignancies (which is a useful summary indicator of good quality of registration processes) has remained very low during the study period[21].

Information was extracted on East of England residents aged ≥40 years diagnosed with oesophageal or gastric cancer (ICD-10 subsite codes C15.0 to C16.9) during the 12-year period between 1995 and 2006, including on tumour site, morphology, patient gender and diagnosis age. We defined five tumour types, according to site and histology (OSCC, OAC, JCA, NCGA, and 'all other' oesophago-gastric cancers), motivated by previous research,[22] and as previously described[23]. Because, OSCC management is substantially different to that of the other oesophagogastric cancers (because of a relatively prominent role of radiotherapy treatment) we excluded OSCC patients from further analysis. More specifically, the following tumour type operational definitions (based on ICD-10 site and ICD-O morphology codes) were used:

• OSCC (defined in order to be excluded from further analysis, as explained above): ICD-10 C150-9 and ICD-O M80703 ('Squamous cell carcinoma', 88% of cases in this category), or M80713, M80723, M80733, M80743, M80943, M81233, M85603).

• OAC: ICD-10 C150-9 and ICD-O M81403 ('Adenocarcinoma', 92% of cases in this category) or M81443, M81453, M82603, M83103, M84803, M84813 and M84903.

• JCA: ICD-10 C160 and ICD-O M81403 ('Adenocarcinoma', 87% of cases in this category) or M81443, M81453, M82103, M82603, M83103, M84803, M84813 and M84903.

• NCGA: ICD-10 C161-9 and ICD-O M81403 ('Adenocarcinoma', 83% of cases in this category) or M81443, M81453, M82103, M82603, M84803, M84813 and M84903.

• 'Other': All other tumours (principally 'Carcinoma', 70% in this category).

We measured patient socioeconomic status with an ecological (i.e. small area) measure. Specifically, patient socioeconomic status was assigned using the Index of Multiple Deprivation (IMD) 2004 deprivation score of their Lower Super Output Area (LSOA) of residence. IMD is composed of different domains, including among others economic, social, education, health and housing indicators, and indicates the level of deprivation of residents of a small area relative to the residents of other small areas[24]. LSOA's are co-terminous small areas with similar socio-demographic characteristics (typically comprising five Census Output Areas with about 1,500 residents). This low level and 'homogenous' aggregation minimises the potential for ecological fallacy (misattribution of individual socioeconomic status using the 'average' socioeconomic profile of a greater sample of individuals)[25]. Quintile groups using the national distribution of LSOA deprivation scores were used in subsequent analysis (groups 1 to 5: 'least' to 'most' deprived respectively).

Information was available on surgical treatment within six months from diagnosis, coded by Registry staff, using the Office for Population and Censuses and Surveys, 4th Revision (OPCS 4) classification system[26]. Curative surgery was defined using appropriate excision surgery codes, and palliative surgery defined as surgical placement of stent or gastrojejunostomy. More specifically, the following operational definitions (based on OPCS 4 codes) were used: *Curative surgery*: G281-3, G288-9: partial excision of stomach (33% of all curative surgery cases); G011-3, G018-9: excision of oesophagus and stomach (25%); G021, G023, G028-9: total excision of oesophagus (8%); G271-5, G278-9: total excision of stomach (17%); G031, G038-9: partial excision of oesophagus (16%); G041-3, G048-9: open extirpation of lesion of oesophagus (1.7%); G291-5, G298: open extirpation of lesion of stomach (1.1%). 1.5% of all patients had more than one of the above curative surgery codes. *Palliative surgery*: G119: Open placement of prosthesis in oesophagus (81%); G331: Bypass of stomach by anastomosis of stomach to jejunum (19%). A patient had both palliative procedures. We analysed these two types of palliative surgery separately in interim analysis, but report the findings in an aggregate fashion, both because disaggregated findings were similar, and because the gastrojejunostomy group was very small (less than a fifth of all palliative surgery). Information was also available on chemotherapy and radiotherapy treatment within six months from diagnosis - whether with a curative or palliative intent.

We examined predictors of use of curative surgery, palliative surgery, chemotherapy, and radiotherapy (patients could belong to one or more of these treatment groups); as well as for 'combined' (within six months from diagnosis) use of both curative surgery and chemotherapy. We did not consider any other combined treatment groups, because of small numbers. For example, within six months from their diagnosis, only 160 (1.1%) patients were treated by both radiotherapy and curative surgery, only 146 (1.0%) by all of curative surgery, chemotherapy and radiotherapy; and only 85 (0.6%) by both curative and palliative surgery.

In univariable analysis, proportions of patients in each treatment group were calculated by age group (three groups, 40-59, 60-74, and ≥75), gender, diagnosis period (four triennial periods, 1995-7, 1998-2000, 2001-3, and 2004-6), deprivation group (1-5), and tumour type. Differences between patient groups were examined with the chi-squared test (for gender or tumour type), or linear regression for ordinal variables (adjusting for age group, diagnosis period or deprivation group as applicable).

In multivariable analysis, predictors of treatment use were examined by logistic regression, with treatment status as the binary dependent variable, and adjusting for gender, age group, diagnosis period, deprivation group and tumour type. We furthermore repeated all analysis by sequentially including in models interaction terms for deprivation and gender, deprivation and age group, and deprivation and diagnosis period.

Stage information was principally limited to 44% (440/1,009) of OAC patients diagnosed between 2004-6, using the 5th Edition of the TNM classification, comprising stages I-IV)[27]. For other cancer types and diagnosis periods, the number of patients with stage information was negligible. For OAC patients diagnosed during 2004-6, we examined predictors of stage ascertainment, and of advanced stage (defined as stages III-IV). We subsequently examined predictors of treatment use, again using multivariable logistic regression analysis, and compared the degree of concordance of the findings relating to staged OAC patients with the findings obtained from the 'all cases' analysis for the same period.

## Results

There were 14,077 patients with relevant oesophagogastric cancers during the 12-year study period (5,112 oesophageal cancers other than OSCC, and 8,965 stomach cancers). 9,653 (69%) of the patients were men. The mean age at diagnosis was significantly greater for women, by +5.6 (75.1 vs. 69.4), +3.5 (72.4 vs. 68.9) and +2.0 (75.0 vs. 73.0) years for OAC, JCA and NCGA respectively.

Overall during the study, 25% of all patients were treated by curative surgery, 8% by palliative surgery, 20% by chemotherapy, 11% by radiotherapy, and 5% by both curative surgery and chemotherapy (see Table S1 in Additional file 1). Between 1995-7 and 2004-6, curative surgery use decreased by about a third (from 28% to 20% of all patients, p < 0.001) and chemotherapy use increased by more than three-fold (from 9% to 30%, p < 0.001). Palliative surgery and radiotherapy use increased significantly but slightly. These associations remained significant in multivariable analysis (See Table S2 in Additional file 2).

**Figure 1:**
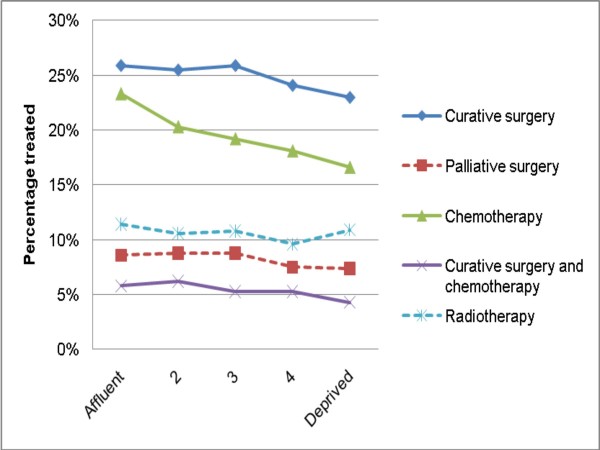
**Percentage of patients treated (with curative surgery, palliative surgery, chemotherapy, curative surgery and chemotherapy in combination, and radiotherapy) by deprivation group**. Continuous lines denote significant and dashed lines non-significant associations in univariable analysis.

**Figure 2:**
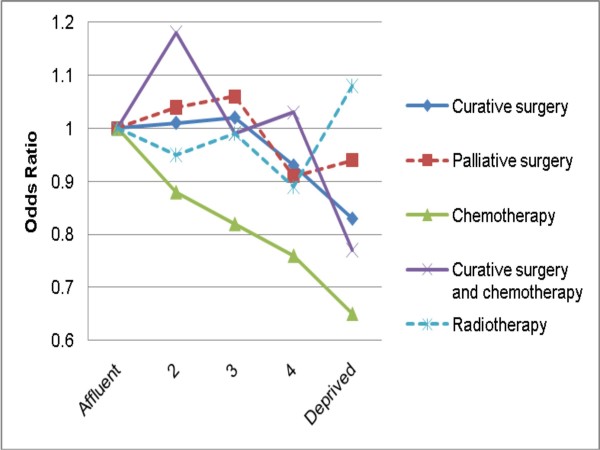
**Odds ratios for treatment use (curative surgery, palliative surgery, chemotherapy, curative surgery and chemotherapy in combination, and radiotherapy) by deprivation group**. Continuous lines denote significant and dashed lines non-significant associations in multivariable analysis adjusting for age group, gender, diagnosis period and tumour type.

In both univariable and multivariable analysis, more deprived patients were less likely to be treated with curative surgery, chemotherapy, and their combined use (Table 1 and Figures 1, 2). These differences were particularly prominent for chemotherapy use. For chemotherapy use there is an apparent linear decrease in frequency of use with increasing deprivation; however, for curative surgery use, there appears to be a (non-linear) 'step' effect, with socioeconomic variation mainly relating to the most (and perhaps the one but most) deprived group. An apparent deprivation gradient for palliative surgery use did not reach significance, whilst no deprivation gradient was apparent in multivariable analysis for radiotherapy use. None of the interaction terms between deprivation and each of gender, age group and diagnosis period were significant, for any of the treatment groups, indicating that the effect of deprivation on treatment use was similar in both sexes, and in any age group and diagnosis period.

**Table 1:** Percentage of patients treated, by treatment and deprivation group

		UNIVARIABLE ANALYSIS			UNADJUSTED LOGISTIC REFRESSION				MULTIVARIABLE ANALYSIS**			
**CURATIVE SURGERY**	**N**	**n**	**%**	p*	**OR**	**95% CI**		**p**	**OR**	**95% CI**		**p**

***Depr. Group cont.***					*0.97*	*0.94*	*1.00*	*0.034*	*0.96*	*0.93*	*0.99*	*0.009*

**'Affluent'**	2,832	733	25.9%	p = 0.034	Ref.				Ref.			
**2**	3,334	850	25.5%		0.98	0.87	1.10		1.01	0.9	1.15	
**3**	3,647	943	25.9%		1.00	0.89	1.12		1.02	0.9	1.15	
**4**	3,053	736	24.1%		0.91	0.81	1.02		0.93	0.82	1.06	
**'Deprived'**	1,211	279	23.0%		0.86	0.73	1.00		0.83	0.70	0.98	

	*14,077*	*3,541*	*25.2%*									
**PALLIATIVE SURGERY**												

***Depr. Group cont.***					*0.96*	*0.91*	*1.00*	*0.074*	*0.96*	*0.91*	*1.00*	*0.076*

**'Affluent'**	2,832	242	8.6%	p = 0.074	Ref.				Ref.			
**2**	3,334	293	8.8%		1.03	0.86	1.23		1.04	0.87	1.25	
**3**	3,647	321	8.8%		1.03	0.87	1.23		1.06	0.89	1.27	
**4**	3,053	230	7.5%		0.87	0.72	1.05		0.91	0.75	1.10	
**'Deprived'**	1,211	90	7.4%		0.86	0.67	1.11		0.94	0.73	1.22	

	*14,077*	*1,176*	*8.4%*									
**CHEMOTHERAPY**												

***Depr. Group cont.***					*0.90*	*0.87*	*0.93*	*< 0.001*	*0.90*	*0.87*	*0.93*	*< 0.001*

**'Affluent'**	2,832	660	23.3%	p < 0.001	Ref.				Ref.			
**2**	3,334	678	20.3%		0.84	0.74	0.95		0.88	0.76	1.01	
**3**	3,647	701	19.2%		0.78	0.69	0.88		0.82	0.71	0.94	
**4**	3,053	552	18.1%		0.73	0.64	0.82		0.76	0.66	0.88	
**'Deprived'**	1,211	201	16.6%		0.65	0.55	0.78		0.65	0.53	0.79	

	*14,077*	*2,792*	*19.8%*									
**CUR. SURGERY +CHEMOTHERAPY**												

***Depr. Group cont.***					*0.93*	*0.88*	*0.99*	*0.023*	*0.94*	*0.88*	*0.99*	*0.045*

**'Affluent'**	2,832	165	5.8%	p = 0.023	Ref.				Ref.			
**2**	3,334	206	6.2%		1.06	0.86	1.31		1.18	0.94	1.47	
**3**	3,647	194	5.3%		0.91	0.73	1.12		0.99	0.79	1.24	
**4**	3,053	161	5.3%		0.90	0.72	1.13		1.03	0.81	1.30	
**'Deprived'**	1,211	52	4.3%		0.73	0.53	1.00		0.77	0.55	1.07	

	*14,077*	*778*	*5.5%*									
**RADIOTHERAPY**												

***Depr. Group cont.***					*0.97*	*0.93*	*1.01*	*0.120*	*0.98*	*0.94*	*1.02*	*0.347*

**'Affluent'**	2,832	324	11.4%	p = 0.120	Ref.				Ref.			
**2**	3,334	354	10.6%		0.92	0.78	1.08		0.95	0.8	1.12	
**3**	3,647	393	10.8%		0.93	0.80	1.09		0.99	0.84	1.17	
**4**	3,053	293	9.6%		0.82	0.70	0.97		0.89	0.75	1.06	
**'Deprived'**	1,211	132	10.9%		0.95	0.76	1.17		1.08	0.87	1.35	

	*14,077*	*1,496*	*10.6%*									

Univariable analysis and unadjusted and adjusted Odds Ratio for treatment use by deprivation group (from multivariable logistic regression models, adjusting for age group, gender, diagnosis period and tumour type).

OR: Odds Ratio; 'Depr.': Deprivation; 'cont'; Continuous (variable); CI: Confidence Interval; 'Cur': Curative.

p*: From linear regression models, entering deprivation group as a continuous variable, as a test for trend.

p**: The full model outputs are presented in Table S2 in Additional file 2

Older patients were significantly less likely to be treated with any of the reviewed treatments except palliative surgery, and these associations remained significant in multivariable analysis.

Men, in univariable analysis, were significantly more likely to be treated by any of the reviewed treatments except palliative surgery. However, in multivarible analysis, men were more likely to be treated only with chemotherapy (p < 0.001), and palliative surgery (but not significantly so, p = 0.092).

Stage information was available for 440 of 1,009 (44%) OAC patients diagnosed during 2004-6. Stage ascertainment was significantly greater for patients <75 (53% vs 32%, p < 0.001) but was not associated with gender or deprivation (data not shown). Advanced stage (i.e. stages III or IV) was not significantly associated with age, gender or deprivation. As observed in 'all patients' analysis, use of curative surgery, and of chemotherapy, decreased with increasing deprivation, but only approaching significance (OR: 0.86, p = 0.134, and OR: 0.88, p = 0.129, respectively). Similarly, there was less frequent chemotherapy use in women, again only approaching significance (OR: 0.64, p = 0.114). Overall, comparing the effects of age, gender and deprivation on use of any of the five reviewed treatments among 'all' and 'staged' OAC patients during 2004-6 (15 comparison pairs in total), eight pairs of observations were concordant for both effect direction and significance level; five were concordant for effect but not for level of significance; and two were discordant (in relation to effect direction).

## Discussion

There were substantial changes over time in the management of over 14,000 oesophageal and stomach cancer patients diagnosed over a 12-year period in the East of England. During the study period, curative surgery use decreased substantially (by over a quarter) and chemotherapy use increased dramatically (by more then three-fold). Adjusting for other variables, more deprived patients were less likely to be treated by curative surgery and by chemotherapy, however this association was much stronger for chemotherapy. Chemotherapy use was also less likely in women.

Deprivation differences in use of curative surgery, although statistically significant, were relatively limited in both absolute and proportional terms (e.g. 26% compared with 23% use, in least and most deprived patients respectively). In contrast, socioeconomic treatment patterning was much stronger for chemotherapy use (e.g. 23% compared with 17% use, in least and most deprived patients respectively). Moreover, although the apparent effect of deprivation status on frequency of chemotherapy use was linear, no such consistent effect was observed for curative surgery use. These observations mean that data limitations (described below) could at least in theory be responsible for the observed socioeconomic difference in use of curative surgery. However, given the substantial effect size of deprivation status on use of chemotherapy, as well as its apparent linear effect, this is less likely to be the case for the observed socioeconomic variation in chemotherapy use, which is more likely to be genuine.

The main strengths of our study are its population-based basis and the fact that it covers a substantial time period and very large number of patients.

The main and most important limitation of our analysis is the lack of information on stage at diagnosis for the great majority of the patients. Population-based survival estimates for oesophageal and stomach cancers have remained poor and changed very little during the study period[6-9]. This would strongly suggest a stable over time distribution of stage among incident cases, that could not account for the observed substantial reduction over time in use of surgery and the dramatic increase in use of chemotherapy. However, lack of stage information poses certain interpretation challenges in relation to the observed socioeconomic differences in use. Several non-UK studies suggest that lower socioeconomic status individuals may have more advanced diagnosis stage[28,29]. This limitation has to be seen in the context of the population-based nature and size of the dataset, and the study period: There is very limited availability of stage information for upper gastrointestinal cancers in population-based datasets covering the same period in England, although this weakness in cancer registration systems is now being rapidly addressed. No significant association between deprivation and stage at presentation was observed among the subgroup of OAC patients for whom stage information was available, but this finding may simply reflect lack of power to detect a significant association, and is best considered as inconclusive. Nevertheless, if more deprived patients in this dataset were presenting at relatively more advanced stage, one could have expected an association between increasing deprivation and increasing use of palliative surgery. No such significant trend was observed, in fact, there was an apparent trend in the opposite direction, which however did not reach significance. Therefore, even if they existed, potential deprivation group differences in stage at presentation are unlikely to fully explain the observed socioeconomic gradients in use of curative surgery and chemotherapy. Previous research also indicates that survival inequalities cannot be fully explained by socioeconomic differences in stage[13,14]. These interpretation challenges should be addressed by further research.

Another limitation is the lack of information on patients' co-morbidity status, which may vary between different oesophago-gastric cancers,[30] and deprivation groups. However, if more deprived patients had a greater co-morbidity burden, one could again expect this to have mainly influenced surgical treatment use (because of the greater anaesthetic risk of patients with higher co-morbidity burden). In contrast, socioeconomic inequalities in both the use of surgery and the use of chemotherapy were observed (and as noted above, socioeconomic variation in use was greater for chemotherapy). These observations would indicate that potential socioeconomic differences in co-morbidity status are unlikely to fully explain the observed deprivation differences in treatment use.

Furthermore, we could not examine whether chemotherapy or radiotherapy were used with a palliative or curative intent. However, the observed increase in combined use of curative surgery and chemotherapy suggests that most of the increase over time in chemotherapy use could be expected to have been in an adjuvant context - although pre-operative chemotherapy will not have actually been followed up by actual surgery in all patients, because of inadequate response.

Treatment status information might have been incomplete (or inaccurate) for some patients - particularly in relation to palliative interventions. However, this would have only introduced non-differential error, and should have not therefore biased the observed findings in relation to socioeconomic or gender variation (or its lack). Such an assumption is both logical and supported by empirical evidence from other 'routine data' sources[31]. Similarly, it is possible that changes during the study period in data quality (including in the degree of treatment status ascertainment) may at least in part be responsible for some of the observed findings. However, there has been a high degree of consistency over time in cancer registration systems used in the UK, where cancer registration has been established over a number of decades. In addition, potential improvements in capturing treatment status could have perhaps explained the observed increase in chemotherapy use but are very unlikely to be responsible for the observed decrease in use of curative surgery. Furthermore, any changes over time in treatment status ascertainment would have been non-differential between patients of different gender and socioeconomic groups, [31] and cannot therefore be responsible for the observed gender and socioeconomic differences in use. For these three reasons, we believe that potential secular changes in data quality are unlikely to be responsible for the observed findings.

We could not examine treatment use beyond six months from diagnosis - however, given the poor prognosis of oesophagogastric cancer, for most patients, treatments that would have been administered beyond that period are likely to have been palliative. Information about whether patients were treated in private hospitals (for all or part of their care) would have been useful. We had no information about other aspects of treatment quality (for example: chemotherapy or radiotherapy dose and regime; treatment timeliness after diagnosis; and adequacy of surgical excision). Information about other quality of care aspects would have made the analysis more informative.

The observed decrease over time in use of curative surgery is consistent with similar trends reported in two recent national studies (which however also included OSCC in analysis)[2,32]. More specifically, an England and Wales audit including information about over 105,000 patients indicated that 'cancer surgery' use in oesophagogastric cancer decreased by nearly a third (from 28% to 20%) between 1998 and 2005,[2] similar to our own findings. Comparing our findings with this study, although we defined tumour categories differently, we also found curative surgery rates being highest for patients with junctional and stomach adenocarcinomas, and relatively lower for OAC patients. Similarly, in Ireland, there was a nearly two-fold reduction in curative surgery use between patients diagnosed in 2000-1 compared with 1994-6[29]. These findings may reflect improved patient selection because of better staging, either by endoscopic ultrasound, and/or CT and PET scanning[2,29].

Previous reports indicate a near doubling in use of 'either chemotherapy or radiotherapy' between 1998 and 2005, from 18% to 34%, without further dis-aggregation of this figure into chemotherapy and radiotherapy[2]. Our findings strongly indicate that the greatest rise in use of non-surgical treatments for (non-OSCC) gastro-oesophageal tumours relates to chemotherapy, probably reflecting increasing peri-operative use in patients judged suitable for curative surgery,[3,4] as also directly observed in our study, with increasing trends in the combined use of chemotherapy and curative surgery.

The findings would support the hypothesis that socioeconomic differences in treatment use may be at least partly responsible for survival inequalities among men with oesophageal and stomach cancer, and, in relation to curative surgery, they are consistent with those from a population-based study in The Netherlands[33].

Deprivation differences in curative surgery and in chemotherapy use were present in both men and women. This provides no insights into reasons for the previously reported better survival in most deprived women with gastric cancer,[7] although potential differences in stage at presentation between the two genders may be responsible. Most of the observed gender differences in treatment use did not persist once the effect of other variables was adjusted for in multivariable analysis, most likely reflecting the fact that women patients were on average older than men. Nevertheless, the observed lower chemotherapy use in women, which persisted even in multivariable analysis adjusting, among other variables, for age, is surprising. As there were no significant gender differences in use of both chemotherapy and curative surgery, the observed differences might principally relate to use of chemotherapy with a palliative intent, however, this hypothesis would require further research.

## Conclusions

Using East of England cancer registration data on the management of oesophago-gastric cancer (other than OSCC) during 1995-2006, we found that curative surgery use decreased and chemotherapy use increased, reflecting improvements in overall quality and appropriateness of care. However, use of chemotherapy, in particular, was less likely in more deprived patients and women. Further research should aim to explore the potential influence of differences in stage at diagnosis and in co-morbidity status on variation in treatment use. It should also encompass other aspects of healthcare quality, for example treatment timeliness.

## Competing interests

The authors declare that they have no competing interests.

## Authors' contributions

GL conceived the original idea for the study, with contributions form all other authors. JB and CG analysed data, and JB, DG and KW collected and quality assured them. MR provided clinical advice and commentary on the methods and on interpretation. All authors interpreted data and have read and approved the final manuscript.

## Pre-publication history

The pre-publication history for this paper can be accessed here:

http://www.biomedcentral.com/1472-6963/9/231/prepub

## Supplementary Material

Additional file 1 **Table S1**. Proportion of patients treated by treatment group, by diagnosis period or other basic characteristic, 1995-2006 (n = 14,077). Click here for file

Additional file 2 **Table S2**. Probability of treatment use by treatment group, 1995-2006 (n = 14,077). Logistic regression models adjusting for gender, age group, deprivation group, diagnosis period and tumour type. Click here for file

## References

[B1] Westlake S Report: Cancer incidence and mortality in the United Kingdom and constituent countries, 2003-05 Office for National Statistics. Health Statistics Quarterly 2008 40 91 7 http://www.statistics.gov.uk/downloads/theme_health/HSQ40CancerUK2003-05.pdf (accessed December 2009) 19093643

[B2] National Oesophago-Gastric Cancer Audit An audit of the care received by people with Oesophago-Gastric Cancer in England and Wales First Annual Report 2008. The Information Centre. NHS http://www.ic.nhs.uk/webfiles/Services/NCASP/Cancer/New%20web%20documents%20(OG)/28010208-NHSIC-OGAuditReport-FV-HR.pdf (accessed December 2009)

[B3] Medical Research Council Oesophageal Cancer Working Group Surgical resection with or without preoperative chemotherapy in oesophageal cancer: randomised controlled trial Lancet 2002 359 9319 1727 33 10.1016/S0140-6736(02)08651-8 12049861

[B4] Cunningham D Allum WH Stenning SP Thompson JN Velde CJ Van de Nicolson M Scarffe JH Lofts FJ Falk SJ Iveson TJ Smith DB Langley RE Verma M Weeden S Chua YJ MAGIC Trial Participants Perioperative chemotherapy versus surgery alone for resectable gastroesophageal cancer N Engl J Med 2006 355 1 11 20 10.1056/NEJMoa055531 16822992

[B5] Stephens MR Blackshaw GR Lewis WG Edwards P Barry JD Hopper NA Allison MC Influence of socio-economic deprivation on outcomes for patients diagnosed with gastric cancer Scand J Gastroenterol 2005 40 1351 7 10.1080/00365520510023666 16334445

[B6] Rachet B Maringe C Nur U Quaresma M Shah A Woods LM Ellis L Walters S Forman D Steward J Coleman MP Population-based cancer survival trends in England and Wales up to 2007: an assessment of the NHS cancer plan for England Lancet Oncol 2009 10 351 69 10.1016/S1470-2045(09)70028-2 19303813

[B7] Mitry E Rachet B Quinn MJ Cooper N Coleman MP Survival from cancer of the stomach in England and Wales up to 2001 Br J Cancer 2008 99 Suppl 1 S16 8 10.1038/sj.bjc.6604574 18813246 PMC2557534

[B8] Mitry E Rachet B Quinn MJ Cooper N Coleman MP Survival from cancer of the oesophagus in England and Wales up to 2001 Br J Cancer 2008 99 Suppl 1 S11 3 10.1038/sj.bjc.6604572 18813240 PMC2557522

[B9] Newnham A Quinn MJ Babb P Kang JY Majeed A Trends in oesophageal and gastric cancer incidence, mortality and survival in England and Wales 1971-1998/1999 Aliment Pharmacol Ther 2003 17 5 655 64 10.1046/j.1365-2036.2003.01520.x 12641514

[B10] Trivers KF De Roos AJ Gammon MD Vaughan TL Risch HA Olshan AF Schoenberg JB Mayne ST Dubrow R Stanford JL Abrahamson P Rotterdam H West AB Fraumeni JF Chow WH Demographic and lifestyle predictors of survival in patients with esophageal or gastric cancers Clin Gastroenterol Hepatol 2005 3 3 225 30 10.1016/S1542-3565(04)00613-5 15765441

[B11] Lyratzopoulos G West CR Williams EM Socioeconomic variation in colon cancer tumour factors associated with poorer prognosis Br J Cancer 2003 89 5 828 30 10.1038/sj.bjc.6601192 12942112 PMC2394465

[B12] Lyratzopoulos G Sheridan GF Michie HR McElduff P Hobbiss JH Absence of socioeconomic variation in survival from colorectal cancer in patients receiving surgical treatment in one health district: cohort study Colorectal Dis 2004 6 6 512 7 10.1111/j.1463-1318.2004.00717.x 15521945

[B13] Woods LM Rachet B Coleman MP Origins of socio-economic inequalities in cancer survival: a review Ann Oncol 2006 17 1 5 19 10.1093/annonc/mdj007 16143594

[B14] Brewster DH Thomson CS Hole DJ Black RJ Stroner PL Gillis CR Relation between socioeconomic status and tumour stage in patients with breast, colorectal, ovarian, and lung cancer: results from four national, population based studies BMJ 2001 322 830 1 10.1136/bmj.322.7290.830 11290637 PMC30560

[B15] Jeffreys M Sarfati D Stevanovic V Tobias M Lewis C Pearce N Blakely T Socioeconomic Inequalities in Cancer Survival in New Zealand:The Role of Extent of Disease at Diagnosis Cancer Epidemiol Biomarkers Prev 2009 18 915 21 10.1158/1055-9965.EPI-08-0685 19223561

[B16] Battersby J Flowers J Harvey I An alternative approach to quantifying and addressing inequity in healthcare provision: access to surgery for lung cancer in the east of England J Epidemiol Community Health 2004 58 7 623 5 10.1136/jech.2003.013391 15194729 PMC1732809

[B17] Nur U Rachet B Parmar MK Sydes MR Cooper N Lepage C Northover JM James R Coleman MP AXIS collaborators No socioeconomic inequalities in colorectal cancer survival within a randomised clinical trial Br J Cancer 2008 2;99 11 1923 8 10.1038/sj.bjc.6604743 PMC2600684 19034284

[B18] Morgan MA Lewis WG Chan DS Burrows S Stephens MR Roberts SA Havard TJ Clark GW Crosby TD Influence of socio-economic deprivation on outcomes for patients diagnosed with oesophageal cancer Scand J Gastroenterol 2007 42 10 1230 7 10.1080/00365520701320471 17852847

[B19] Department of Health The NHS Cancer plan:a plan for investment, a plan for reform http://www.dh.gov.uk/en/Publicationsandstatistics/Publications/PublicationsPolicyAndGuidance/DH_4009609 (accessed December 2009)

[B20] Department of Health Cancer Reform Strategy http://www.dh.gov.uk/en/Publicationsandstatistics/Publications/PublicationsPolicyAndGuidance/DH_081006 (accessed December 2009)

[B21] UKACR Quality and Performance Indicators 2007: Final. Table 6 http://82.110.76.19/quality/UKACR%20report2009_final.xls (accessed December 2009)

[B22] Botterweck AA Schouten LJ Volovics A Dorant E Brandt PA van Den Trends in incidence of adenocarcinoma of the oesophagus and gastric cardia in ten European countries Int J Epidemiol 2000 29 4 645 54 10.1093/ije/29.4.645 10922340

[B23] Gajperia C Barbiere J Greenberg D Wright K Lyratzopoulos G Recent incidence trends and socio-demographic features of oesophageal and gastric cancer types in an English region Aliment Pharmacol Ther 2009 30 8 873 80 10.1111/j.1365-2036.2009.04100.x 19624549

[B24] Communities and Local Government Indices of Deprivation 2007 http://www.communities.gov.uk/communities/neighbourhoodrenewal/deprivation/deprivation07/(accessed December 2009)

[B25] Woods LM Rachet B Coleman MP Choice of geographic unit influences socioeconomic inequalities in breast cancer survival Br J Cancer 2005 92 7 1279 82 10.1038/sj.bjc.6602506 15798765 PMC2361971

[B26] NHS Connecting for Health OPCS-4 Intervention Classification http://www.connectingforhealth.nhs.uk/systemsandservices/data/clinicalcoding/codingstandards/opcs4 (accessed December 2009)

[B27] Sobin LH Wittekind Ch editors International Union Against Cancer (UICC) TNM classification of malignant tumors 1997 5 New York: John Wiley & Sons, Inc

[B28] Halpern MT Ward EM Pavluck AL Schrag NM Bian J Chen AY Association of insurance status and ethnicity with cancer stage at diagnosis for 12 cancer sites:a retrospective analysis Lancet Oncol 2008 9 3 222 31 10.1016/S1470-2045(08)70032-9 18282806

[B29] Clegg LX Reichman ME Miller BA Hankey BF Singh GK Lin YD Goodman MT Lynch CF Schwartz SM Chen VW Bernstein L Gomez SL Graff JJ Lin CC Johnson NJ Edwards BK Impact of socioeconomic status on cancer incidence and stage at diagnosis: selected findings from the surveillance, epidemiology, and end results: National Longitudinal Mortality Study Cancer Causes Control 2008 20 417 35 10.1007/s10552-008-9256-0 19002764 PMC2711979

[B30] Koppert LB Janssen-Heijnen ML Louwman MW Lemmens VE Wijnhoven BP Tilanus HW Coebergh JW Comparison of comorbidity prevalence in oesophageal and gastric carcinoma patients: a population-based study Eur J Gastroenterol Hepatol 2004 16 7 681 8 10.1097/01.meg.0000108331.52416.f1 15201582

[B31] Lyratzopoulos G Heller RF Hanily M Lewis PS Risk factor measurement quality in primary care routine data was variable but nondifferential between individuals J Clin Epidemiol 2008 61 261 267 10.1016/j.jclinepi.2007.05.020 18226749

[B32] Cronin-Fenton DP Sharp L Carsin AE Comber H Patterns of care and effects on mortality for cancers of the oesophagus and gastric cardia: a population-based study Eur J Cancer 2007 43 3 565 75 10.1016/j.ejca.2006.10.011 17140789

[B33] van Vliet EP Eijkemans MJ Steyerberg EW Kuipers EJ Tilanus HW Gaast A van der Siersema PD The role of socio-economic status in the decision making on diagnosis and treatment of oesophageal cancer in The Netherlands Br J Cancer 2006 95 9 1180 5 10.1038/sj.bjc.6603374 17031405 PMC2360583

