# Supplementary material for: Trends and variation in the management of oesophagogastric cancer patients: a population-based survey
Source: BMC Health Serv Res. 2009 Dec 15;9:231. doi: 10.1186/1472-6963-9-231 (PMC2813235; doi:10.1186/1472-6963-9-231)
Supplement: Additional file 1 — Table S1. Proportion of patients treated by treatment group, by diagnosis period or other basic characteristic, 1995-2006 (n = 14,077). [file 1472-6963-9-231-S1.DOC]

**Table S1 in Additional file 1. Proportion of patients treated by treatment group, by diagnosis period** or other basic characteristic, 1995-2006 (n=14,077)

|  |  |  | **Curative surgery** | | | **Chemotherapy** | | | **Both curative surgery and chemotherapy** | | | **Palliative Surgery** | | | **Radiotherapy** | | |
| --- | --- | --- | --- | --- | --- | --- | --- | --- | --- | --- | --- | --- | --- | --- | --- | --- | --- |
|  |  |  | **(n=3,541)** | | | **(n=2,792)** | | | **(n=778)** | | | **(n=1,176)** | | | **(n=1,496)** | | |
|  | **Categories** | **N** | **n** | **%** | **p** | **n** | **%** | **p** | **n** | **%** | **p** | **n** | **%** | **p** | **n** | **%** | **p** |
| **Diagnosis** | **1995-7** | 3,764 | 1,044 | 27.7% | p*<0.001 | 353 | 9.4% | p*<0.001 | 88 | 2.3% | p*<0.001 | 255 | 6.8% | p*<0.001 | 333 | 8.9% | p*=0.001 |
| **period** | **1998-2000** | 3,558 | 981 | 27.6% |  | 545 | 15.3% |  | 148 | 4.2% |  | 299 | 8.4% |  | 387 | 10.9% |  |
|  | **2001-3** | 3,415 | 838 | 24.5% |  | 905 | 26.5% |  | 252 | 7.4% |  | 301 | 8.8% |  | 407 | 11.9% |  |
|  | **2004-6** | 3,340 | 678 | 20.3% |  | 989 | 29.6% |  | 290 | 8.7% |  | 321 | 9.6% |  | 369 | 11.1% |  |
|  | *All eras* | *14,077* | *3,541* | *25.2%* |  | *2,792* | *19.8%* |  | *778* | *5.5%* |  | *1,176* | *8.4%* |  | *1,496* | *10.6%* |  |
| **Age group** | **40-59** | 1,834 | 753 | 41.1% | p*<0.001 | 881 | 48.0% | p*<0.001 | 298 | 16.3% | p*<0.001 | 133 | 7.3% | p*=0.004 | 269 | 14.7% | p*<0.001 |
|  | **60-74** | 5,352 | 1,765 | 33.0% |  | 1,521 | 28.4% |  | 411 | 7.7% |  | 421 | 7.9% |  | 679 | 12.7% |  |
|  | ≥**75** | 6,891 | 1,023 | 14.9% |  | 390 | 5.7% |  | 69 | 1.0% |  | 622 | 9.0% |  | 548 | 8.0% |  |
|  | *All groups* | *14,077* | *3,541* | *25.2%* |  | *2,792* | *19.8%* |  | *778* | *5.5%* |  | *1,176* | *8.4%* |  | *1,496* | *10.6%* |  |
| **Gender** | **Male** | 9,653 | 2,609 | 27.0% | p<0.001 | 2,213 | 22.9% | p*<0.001 | 615 | 6.4% | p<0.001 | 814 | 8.4% | p=0.619 | 1,137 | 11.8% | p<0.001 |
|  | **Female** | 4,424 | 932 | 21.1% |  | 579 | 13.1% |  | 163 | 3.7% |  | 362 | 8.2% |  | 359 | 8.1% |  |
|  | *Persons* | *14,077* | *3,541* | *25.2%* |  | *2,792* | *19.8%* |  | *778* | *5.5%* |  | *1,176* | *8.4%* |  | *1,496* | *10.6%* |  |
| **Deprivation** | **‘Affluent’** | 2,832 | 733 | 25.9% | p*=0.034 | 660 | 23.3% | p*<0.001 | 165 | 5.8% | p*=0.023 | 242 | 8.6% | p*=0.074 | 324 | 11.4% | p*=0.120 |
| **group** | **2** | 3,334 | 850 | 25.5% |  | 678 | 20.3% |  | 206 | 6.2% |  | 293 | 8.8% |  | 354 | 10.6% |  |
|  | **3** | 3,647 | 943 | 25.9% |  | 701 | 19.2% |  | 194 | 5.3% |  | 321 | 8.8% |  | 393 | 10.8% |  |
|  | **4** | 3,053 | 736 | 24.1% |  | 552 | 18.1% |  | 161 | 5.3% |  | 230 | 7.5% |  | 293 | 9.6% |  |
|  | **‘Deprived’** | 1,211 | 279 | 23.0% |  | 201 | 16.6% |  | 52 | 4.3% |  | 90 | 7.4% |  | 132 | 10.9% |  |
|  | *All groups* | *14,077* | *3,541* | *25.2%* |  | *2,792* | *19.8%* |  | *778* | *5.5%* |  | *1,176* | *8.4%* |  | *1,496* | *10.6%* |  |
| **Tumour** | **OAC** | 3,726 | 844 | 22.7% | p<0.001 | 1,003 | 26.9% | p<0.001 | 265 | 7.1% | p<0.001 | 565 | 15.2% | p<0.001 | 811 | 21.8% | p<0.001 |
| **type** | **JCA** | 2,108 | 798 | 37.9% |  | 701 | 33.3% |  | 269 | 12.8% |  | 212 | 10.1% |  | 291 | 13.8% |  |
|  | **NCGA** | 4,765 | 1,644 | 34.5% |  | 776 | 16.3% |  | 203 | 4.3% |  | 201 | 4.2% |  | 162 | 3.4% |  |
|  | **All other** | 3,478 | 255 | 7.3% |  | 312 | 9.0% |  | 41 | 1.2% |  | 198 | 5.7% |  | 232 | 6.7% |  |
|  | *All types* | *14,077* | *3,541* | *25.2%* |  | *2,792* | *19.8%* |  | *778* | *5.5%* |  | *1,176* | *8.4%* |  | *1,496* | *10.6%* |  |

OAC: Oesophageal Adeno-Carcinoma; JCA: Junctional / Cardia Adenocarcinoma; NCGA: Non-Cardia Gastric Adenocarcinoma. p*: from linear regression models, adjusting for age group, diagnosis period and deprivation group as applicable
