# Supplementary material for: Trends and variation in the management of oesophagogastric cancer patients: a population-based survey
Source: BMC Health Serv Res. 2009 Dec 15;9:231. doi: 10.1186/1472-6963-9-231 (PMC2813235; doi:10.1186/1472-6963-9-231)
Supplement: Additional file 2 — Table S2. Probability of treatment use by treatment group, 1995-2006 (n = 14,077). Logistic regression models adjusting for gender, age group, deprivation group, diagnosis period and tumour type. [file 1472-6963-9-231-S2.DOC]

**Table S2 in Additional file 2. Probability of treatment use by treatment group, 1995-2006 (n=14,077). Logistic regression models adjusting for gender, age group, deprivation group, diagnosis period and tumour type**.

|  | **Curative surgery** | | | | **Palliative Surgery** | | | | **Chemotherapy** | | | | **Both curative surgery and chemotherapy** | | | | **Radiotherapy** | | | |
| --- | --- | --- | --- | --- | --- | --- | --- | --- | --- | --- | --- | --- | --- | --- | --- | --- | --- | --- | --- | --- |
|  | **OR** | **95% CI** | | **p** | **OR** | **95% CI** | | **p** | **OR** | **95% CI** | | **p** | **OR** | **95% CI** | | **p** | **OR** | **95% CI** | | **p** |
| **GENDER** |  |  |  |  |  |  |  |  |  |  |  |  |  |  |  |  |  |  |  |  |
| **Male** | Ref. |  |  |  | Ref. |  |  |  | Ref. |  |  |  | Ref. |  |  |  | Ref. |  |  |  |
| **Female** | 0.94 | 0.86 | 1.03 | 0.202 | 1.12 | 0.98 | 1.29 | 0.092 | 0.76 | 0.68 | 0.85 | <0.001 | 0.92 | 0.76 | 1.11 | 0.398 | 0.9 | 0.79 | 1.03 | 0.12 |
| **AGE GROUP** |  |  |  |  |  |  |  |  |  |  |  |  |  |  |  |  |  |  |  |  |
| ***Age group cont.*** | *0.46* | *0.43* | *0.48* | *<0.001* | *1.15* | *1.05* | *1.25* | *0.002* | *0.25* | *0.23* | *0.27* | *<0.001* | *0.27* | *0.25* | *0.30* | *<0.001* | *0.7* | *0.65* | *0.75* | *<0.001* |
| **40-59** | Ref. |  |  |  | Ref. |  |  |  | Ref. |  |  |  | Ref. |  |  |  | Ref. |  |  |  |
| **60-74** | 0.65 | 0.58 | 0.73 |  | 1.23 | 1 | 1.51 |  | 0.44 | 0.39 | 0.5 |  | 0.46 | 0.39 | 0.54 |  | 0.97 | 0.83 | 1.14 |  |
| ≥**75** | 0.25 | 0.22 | 0.28 |  | 1.62 | 1.33 | 1.98 |  | 0.07 | 0.06 | 0.08 |  | 0.06 | 0.05 | 0.08 |  | 0.67 | 0.57 | 0.79 |  |
| **DEPR. GROUP** |  |  |  |  |  |  |  |  |  |  |  |  |  |  |  |  |  |  |  |  |
| ***DG cont.*** | *0.96* | *0.93* | *0.99* | *0.009* | *0.96* | *0.91* | *1* | *0.076* | *0.9* | *0.87* | *0.93* | *<0.001* | *0.94* | *0.88* | *0.99* | *0.045* | *0.98* | *0.94* | *1.02* | *0.347* |
| **‘Affluent’** | Ref. |  |  |  | Ref. |  |  |  | Ref. |  |  |  | Ref. |  |  |  | Ref. |  |  |  |
| **2** | 1.01 | 0.9 | 1.15 |  | 1.04 | 0.87 | 1.25 |  | 0.88 | 0.76 | 1.01 |  | 1.18 | 0.94 | 1.47 |  | 0.95 | 0.8 | 1.12 |  |
| **3** | 1.02 | 0.9 | 1.15 |  | 1.06 | 0.89 | 1.27 |  | 0.82 | 0.71 | 0.94 |  | 0.99 | 0.79 | 1.24 |  | 0.99 | 0.84 | 1.17 |  |
| **4** | 0.93 | 0.82 | 1.06 |  | 0.91 | 0.75 | 1.1 |  | 0.76 | 0.66 | 0.88 |  | 1.03 | 0.81 | 1.3 |  | 0.89 | 0.75 | 1.06 |  |
| **‘Deprived’** | 0.83 | 0.7 | 0.98 |  | 0.94 | 0.73 | 1.22 |  | 0.65 | 0.53 | 0.79 |  | 0.77 | 0.55 | 1.07 |  | 1.08 | 0.87 | 1.35 |  |
| **DIAGNOSIS PERIOD** |  |  |  |  |  |  |  |  |  |  |  |  |  |  |  |  |  |  |  |  |
| ***Diagnosis era cont.*** | *0.84* | *0.81* | *0.88* | *<0.001* | *1.12* | *1.07* | *1.19* | *<0.001* | *1.72* | *1.65* | *1.8* | *<0.001* | *1.58* | *1.47* | *1.69* | *<0.001* | *1.09* | *1.04* | *1.15* | *<0.001* |
| **1995-7** | Ref. |  |  |  | Ref. |  |  |  | Ref. |  |  |  | Ref. |  |  |  | Ref. |  |  |  |
| **1998-2000** | 0.91 | 0.82 | 1.02 |  | 1.19 | 1 | 1.42 |  | 1.77 | 1.52 | 2.07 |  | 1.72 | 1.31 | 2.27 |  | 1.18 | 1.01 | 1.39 |  |
| **2001-3** | 0.73 | 0.65 | 0.82 |  | 1.2 | 1 | 1.43 |  | 3.84 | 3.32 | 4.44 |  | 3.03 | 2.35 | 3.91 |  | 1.24 | 1.06 | 1.45 |  |
| **2004-6** | 0.58 | 0.51 | 0.65 |  | 1.28 | 1.07 | 1.52 |  | 4.8 | 4.15 | 5.55 |  | 3.82 | 2.98 | 4.91 |  | 1.1 | 0.93 | 1.29 |  |
| **TUMOUR TYPE** |  |  |  |  |  |  |  |  |  |  |  |  |  |  |  |  |  |  |  |  |
| **OAC** | Ref. |  |  |  | 4.25 | 3.59 | 5.04 | <0.001 | 1.44 | 1.28 | 1.62 | <0.001 | 1.26 | 1.04 | 1.54 | 0.019 | 7.52 | 6.3 | 8.97 | <0.001 |
| **JAC** | 2.05 | 1.82 | 2.31 | <0.001 | 2.72 | 2.22 | 3.33 | <0.001 | 1.85 | 1.62 | 2.11 | <0.001 | 2.32 | 1.9 | 2.83 | <0.001 | 4.2 | 3.43 | 5.14 | <0.001 |
| **NCGA** | 2.12 | 1.91 | 2.34 | <0.001 | Ref. |  |  |  | Ref. |  |  |  | Ref. |  |  |  | Ref. |  |  |  |
| **All other** | 0.31 | 0.27 | 0.37 | <0.001 | 1.33 | 1.09 | 1.63 | 0.005 | 0.58 | 0.5 | 0.67 | <0.001 | 0.31 | 0.22 | 0.44 | <0.001 | 2.15 | 1.75 | 2.65 | <0.001 |

OR: Odds Ratio; ‘Depr.’: Deprivation; ‘cont’; Continuous (variable); CI: Confidence Interval; OAC: Oesophageal Adeno-Carcinoma; JCA: Junctional / Cardia Adenocarcinoma; NCGA: Non-Cardia Gastric Adenocarcinoma.
